# State of the art of the Fontan strategy for treatment of univentricular heart disease

**DOI:** 10.12688/f1000research.13792.1

**Published:** 2018-06-27

**Authors:** Jelle P. G. van der Ven, Eva van den Bosch, Ad J.C.C. Bogers, Willem A. Helbing

**Affiliations:** 1 Department of Pediatrics, Division of Pediatric Cardiology, Erasmus MC-Sophia Children’s Hospital, Rotterdam, Netherlands; 2 Netherlands Heart Institute, Utrecht, Netherlands; 3 Department of Cardiothoracic Surgery, Erasmus MC, Rotterdam, Netherlands

**Keywords:** Fontan Procedure, Total cavopulmonary connection, Congenital heart defects, Single ventricle, Pediatrics, Mortality, Morbidity, Re-interventions

## Abstract

In patients with a functionally univentricular heart, the Fontan strategy achieves separation of the systemic and pulmonary circulation and reduction of ventricular volume overload. Contemporary modifications of surgical techniques have significantly improved survival. However, the resulting Fontan physiology is associated with high morbidity. In this review, we discuss the state of the art of the Fontan strategy by assessing survival and risk factors for mortality. Complications of the Fontan circulation, such as cardiac arrhythmia, thromboembolism, and protein-losing enteropathy, are discussed. Common surgical and catheter-based interventions following Fontan completion are outlined. We describe functional status measurements such as quality of life and developmental outcomes in the contemporary Fontan patient. The current role of drug therapy in the Fontan patient is explored. Furthermore, we assess the current use and outcomes of mechanical circulatory support in the Fontan circulation and novel surgical innovations. Despite large improvements in outcomes for contemporary Fontan patients, a large burden of disease exists in this patient population. Continued efforts to improve outcomes are warranted. Several remaining challenges in the Fontan field are outlined.

## Introduction

Functionally univentricular congenital heart disease (CHD), in which only one ventricle is fully developed, poses a complex clinical problem. Estimates of the incidence of this disease entity range from 0.08 to 0.4 per 1,000 births ^1– 3^. Functionally univentricular CHD entails different morphological diagnoses, the most common of which are hypoplastic left heart syndrome (HLHS) (25 to 67% of functionally univentricular hearts), tricuspid atresia (15 to 24%), and double inlet left ventricle (14 to 18%) ^1, 3– 5^. It is estimated that currently there are about 22,000 patients in Europe and about 50,000 in the US ^6^. Recent advancements in prenatal screening have increased the rates of prenatal diagnosis and possibly termination of pregnancy in patients with univentricular hearts ^1, 7^. Despite the low incidence, improvements in treatment have reduced the mortality to the point where a large number of patients survive into adulthood.

Palliation can be achieved with the Fontan strategy. A series of operations is performed to palliate the adverse effects of a univentricular heart. The Fontan strategy refers to the landmark surgery for tricuspid atresia by Fontan and Baudet ^8^. In the “early days” of this procedure, it was attempted to replace the function of the right ventricle with the right atrium by connecting the right atrium to the pulmonary artery. Although short-term results were unprecedented, this strategy caused dilation of the right atrium, resulting in arrhythmia and thromboembolism due to sluggish blood flow ^9^. Modifications of this surgery are referred to as atriopulmonary connections (APCs). In a later era, de Leval *et al*. found that atrial contractions did not contribute significant power to the APC circuit and proposed the intra-atrial lateral tunnel (ILT), a transatrial connection using an intra-atrial baffle connecting the inferior caval vein to the pulmonary artery in a more energetically favorable manner ^10^. Currently, most centers employ an extracardiac conduit (ECC), a prosthetic conduit that bypasses the atrium completely. Both ILT and ECC are referred to as total cavopulmonary connection (TCPC) Fontan modifications.

**Figure 1.**
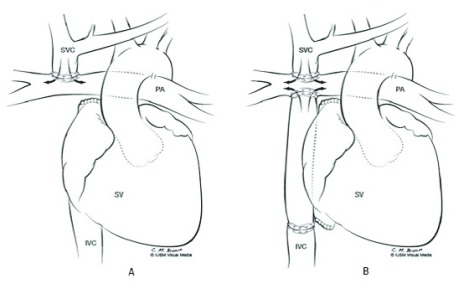
Illustration of the anatomic relationships following partial cavopulmonary connection ( **A**) and total cavopulmonary connection ( **B**) palliation. IVC, inferior vena cava; PA, pulmonary artery; SV, single ventricle; SVC, superior vena cava. This figure has been reproduced with permission from Kerlo *et al*. and Springer Nature ^11^.

A Fontan circuit was originally created in a single surgical setting. This resulted in relatively high mortality. A staged TCPC, in which a series of operations is performed at different ages, is the current standard of care. These operations are tailored to the individual anatomy of the patient. First, the single ventricle (SV) needs to be connected to the aorta, which may require extensive surgery, such as the Norwood procedure for HLHS. At about 3 to 6 months of age, a partial cavopulmonary connection (PCPC), connecting the superior caval vein to the pulmonary artery (that is, bidirectional Glenn procedure), is performed. Completion of the TCPC is usually performed between 18 months and 4 years of age ^4^. The connections and circulatory pattern after these operations are illustrated in Figure 1.

These patients require a lifetime of highly specialized care and significant health-care resources. In Oceania, the mean hospital costs across all stages of palliation are about $200,000 per patient ^12^. Following Fontan palliation, hospital admission rates for patients are eight times higher than for the general population ^13^, and both length of stay and hospital costs are higher compared with those of other CHDs, including tetralogy of Fallot ^14– 16^.

### Physiology

**Figure 2.**
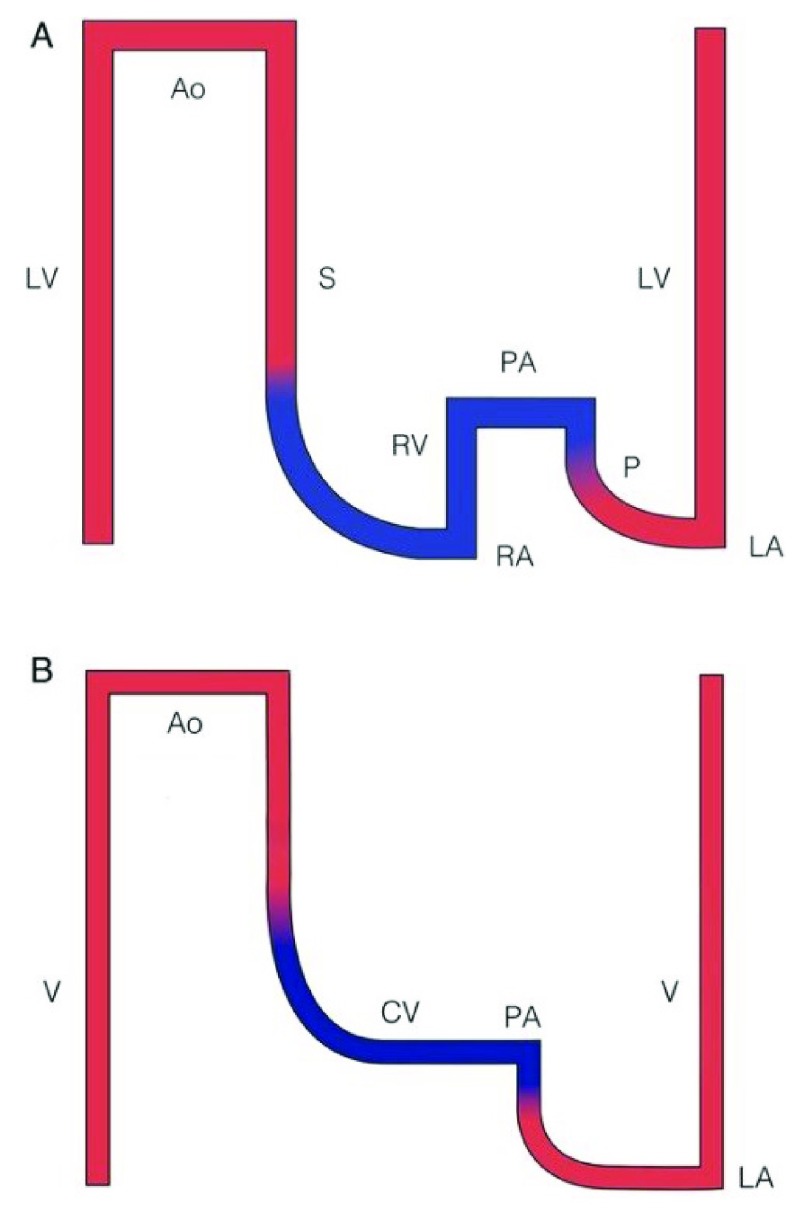
Scheme of pressures in the normal circulation ( **A**) and the Fontan circulation ( **B**). This scheme illustrates the effects of the lack of a prepulmonary pump in the Fontan physiology. Red represents oxygenated blood and blue represents deoxygenated blood. Ao, aorta; CV, caval veins; LA, left atrium; LV, left ventricle; P, pulmonary circulation; PA, pulmonary artery; RA, right atrium; RV, right ventricle; S, systemic circulation; V, single ventricle. This figure has been reproduced with permission from Gewillig and Brown and the British Medical Journal Publishing Group Ltd ^20^.

In unpalliated univentricular CHD, cyanosis occurs because of mixing of saturated and unsaturated blood in the heart. The SV is also exposed to volume overload as it drains both systemic and pulmonary venous return at the same time. The Fontan strategy reduces volume overload and restores normoxemia. Following PCPC surgery, some volume unloading of the SV is achieved. Following TCPC, the volume load of the SV is further reduced ^17^. Furthermore, after TCPC, the systemic and pulmonary blood flows are connected in series rather than parallel as before this strategy has been deployed. This comes at the expense of the lack of a ventricle supplying energy to the pulmonary circulation. This is illustrated in Figure 2. The SV provides the energy needed to attain blood flow through the systemic as well as the pulmonary vascular bed and is subjected to increased afterload. After TCPC, central venous pressures are higher than normal. Pulsatility in the pulmonary artery is mostly lost and there is preload insufficiency of the SV. This highly abnormal circulation is called the Fontan circulation. The resulting physiology has been referred to as a “Fontan paradox”, where systemic venous pressure is high in the presence of relative pulmonary artery hypotension ^18^. This might augment lymphatic outflow and impede lymphatic inflow from the thoracic duct. Several complications of the Fontan strategy have been linked to abnormalities in lymphatic drainage. Because of the multiple inherent hemodynamic challenges of the Fontan circulation, it is generally considered a palliative procedure ^19^.

The aim of this article is to provide an overview of current outcomes, treatment options, and remaining challenges to improve outlook for patients with univentricular heart disease.

## State of the art

### Overall survival

**Figure 3.**
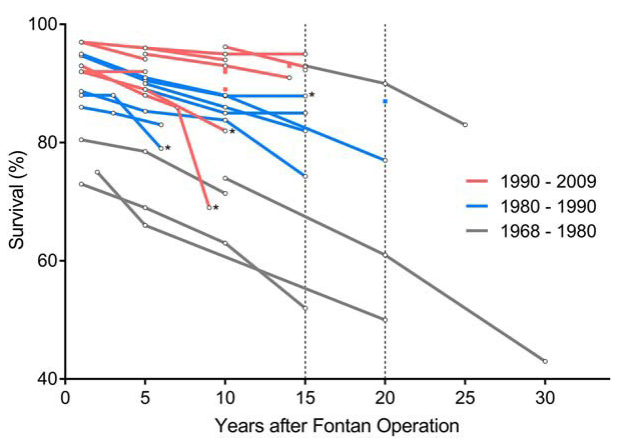
Survival following Fontan completion. Each line represents a study assessing survival at multiple time points and is colored by surgical era. Dots represent Kaplan-Meier survival estimates. Studies marked with an asterisk show survival curves for death, transplant, or Fontan revision; others show survival curves for only death. This figure has been reproduced with permission from Kverneland *et al*. and John Wiley and Sons ^21^.

Survival following the Fontan procedure has increased dramatically in the past few decades. We will discuss data from recently published reports of large cohorts with long follow-up intervals. An overview of studies assessing survival is presented in Figure 3, obtained from Kverneland *et al*. ^21^.

In a recent study from Oceania, perioperative mortality decreased from 8% between 1975 and 1990 to 1% in 2001–2010 ^22^. In this cohort, early Fontan takedown occurred in 2% of patients. Ten-year survival among patients discharged with a Fontan circulation was 89% following APC and 97% for both ECC and ILT ^23^. Survival at 25 years was 76%. This group was composed only of APC patients.

In a retrospective study from the Mayo Clinic of 40 years and 1,052 Fontan patients, overall survival rates were 74% at 10 years, 61% at 20 years, and 43% at 30 years ^24^. Survival was significantly higher in later surgical eras, and 10-year survival was 95% for patients operated on after 2001. Interestingly, patients operated on with the ECC technique showed better overall survival over ILT. It should be taken into account that patients with cardiac defects with worse prognosis were more frequently operated on using the ILT technique.

A Danish national registry described outcomes for SV patients from 1977 to 2009 ^1^. Fifty percent of patients died before Fontan completion. Overall survival improved in later birth eras. Five-year survival for any univentricular CHD increased from 22% in 1977–1989 to 51% in 2000–2009.

One should note that follow-up studies after Fontan procedures reflect results of an earlier surgical era and do not necessarily represent the outlook of Fontan patients undergoing surgery in the current era.

### Determinants of survival

Several factors have been associated with survival after the Fontan procedure. Of preoperative factors, male gender ^23^ and specific CHD diagnosis, most strikingly for HLHS ^24^, have been associated with worse long-term survival. Perioperative risk factors for mortality include APC type procedure, earlier surgical era, older age at procedure, concomitant valve replacement, and prolonged postoperative pleural effusion ^25^. Postoperative factors that affect survival include elevated central venous pressure and lower arterial saturation ^26^; imaging-derived parameters such as lower global longitudinal strain on echocardiography and higher end-diastolic volume measured by magnetic resonance imaging (MRI) ^25^; peak heart rate and peak oxygen uptake during cardiopulmonary exercise testing ^25, 27, 28^; and serum levels of sodium, creatinine, and brain natriuretic peptide ^25, 29, 30^.

### Cardiac complications

Although survival following Fontan completion has increased over time, the Fontan strategy has been associated with important morbidity, most likely related to extensive surgical procedures and the highly abnormal circulatory state after these procedures. In recent cohorts, event-free survival has ranged from 59 to 81% at 15 years ^23, 31^.

The most commonly reported event is cardiac arrhythmia. Supraventricular tachycardia (SVT) contributes significantly to late mortality in Fontan patients ^32^. SVT has been associated with Fontan type; the highest risk is for APC type, followed by ILT, and the lowest is for ECC ^23, 33^. Results may have improved for the ILT technique after introduction of the prosthetic modification ^34^. The incidence of SVT increases during follow-up, and after 20 years of follow-up, 10 to 60% of patients have experienced some form of SVT ^34, 35^.

Failure of the Fontan circulation can occur even in long-standing uncomplicated Fontan circulations. The definition of Fontan failure varies but generally includes excessive constitutional limitations of the Fontan physiology, abnormal parameters of hemodynamic function, poor functional status, or the presence of Fontan sequelae ^36^. Fontan failure occurs in 2 to 13% of patients, depending on definitions and follow-up period ^36^. A 56% 25-year freedom of Fontan failure for APC connections has been reported ^23^.

### Extracardiac complications

***Abnormalities of coagulation.*** Blood flow can be slow in the Fontan circuit because of the absence of a prepulmonary pump. This promotes coagulation. Furthermore, coagulation factor abnormalities have been described in the SV patient, even before PCPC palliation ^37^. Thromboembolic events are more common following APC surgery than TCPC ^37^. The prevalence of thrombi is particularly high in those who develop atrial arrhythmias ^38^. Up to 65% of thrombi are detected within the first year following TCPC (early thromboembolic events) ^39^. Early thromboembolic events probably relate to perioperative factors, such as extracorporeal membrane oxygenation (ECMO) use and altered hemodynamics. Beyond 10 years after Fontan completion, incidence of late thromboembolism steadily increases ^39^. Reports of the incidence of thromboembolism are complicated by different definitions and methods of detection. Studies report incidences of late thromboembolism of between 1 and 25% ^31, 40^. Silent thrombi are detected with transesophageal echocardiography in up to 33% of patients ^41^. Thromboembolic events have been reported to account for 8% of Fontan deaths ^42^.

Liver function abnormalities, cirrhosis, and even hepatocellular carcinoma have been reported following Fontan surgery ^43^. The elevated systemic venous pressures encountered in the Fontan circulation lead to chronic venous congestion in the liver. However, the exact mechanism for Fontan-associated liver disease remains unknown, as does how best to monitor progression ^44, 45^. The American College of Cardiology recently provided a position statement on the subject, advising laboratory and imaging screening at least every 3–5 years in children and at least every 1–3 years in adult Fontan patients ^46^. Preventive strategies need to be developed.

Protein-losing enteropathy (PLE) is a devastating complication of the Fontan circulation in which loss of protein in the gastrointestinal tract occurs, leading to low albumin levels, edema, pleural effusions, and ascites. Incidences of between 3 and 29% have been reported ^24, 31, 35, 47^. It is thought to be caused, in part, by impairments in the lymphatic drainage. Hepatoduodenal lymphatic connections exist in some patients as a normal anatomic variant, which might induce competition in lymphatic flow in the presence of elevated venous pressure, diverting lymphatic flow to the gastrointestinal tract.

Plastic bronchitis is a serious pulmonary complication of the Fontan circulation in which large gelatinous casts are formed in the airways. It is thought to be related to abnormal lymphatic drainage directly into the airways, resulting in cast formation. Reported incidences range from 0.5 to 4% ^48– 50^.

Chronic kidney disease is estimated to be present in up to 50% of adult Fontan patients and is associated with adverse outcomes ^30, 51^. With the increasing availability of cystatin C-determined glomerular filtration rate (GFR), a muscle mass-independent estimate, the prevalence of clinically significant renal dysfunction appears to be lower ^52^. GFR estimates based on serum creatinine concentrations probably overestimate renal function in Fontan patients ^51^.

Psychological, psychiatric, and cognitive defects have been described in Fontan patients. Cerebral MRI has shown morphological differences in some cerebral structures of Fontan patients compared with those of healthy controls ^53, 54^. Interestingly, the pituitary gland, supplied by a portal venous system similar to the liver, appears to be enlarged following Fontan surgery ^55^. The relevance of this possible congestion on the endocrine system remains unclear.

### Re-interventions

Many patients require additional surgical and catheter-based interventions following Fontan completion. Twenty-year freedom of re-operation following TCPC procedures in recent eras ranges from 86 to 92% ^56^. In older cohorts, higher re-operation rates have been reported ^24, 57, 58^. The most common surgical re-intervention procedures, in order of incidence, are pacemaker implantation in 9 to 23% of patients ^23, 24^, Fontan revision or conversion in 3 to 18% of patients ^9, 24, 59^, and atrioventricular (AV) valve repair in 1 to 14% of patients ^24, 59, 60^.

Fontan conversion, from APC to TCPC, can improve functional status and exercise tolerance in the failing Fontan circulation ^61^. AV valve repair after Fontan completion is considered in patients with moderate to severe regurgitation, but survival following successful repair remains inferior to that of patients without prior AV regurgitation ^62, 63^.

For several reasons, re-interventions by catheter may be required. A fenestration in the atrial tunnel, inducing a right-to-left atrial shunt, can be created during surgery to decrease systemic venous pressure, increase ventricular preload, and improve cardiac output at the cost of lower arterial saturation. This fenestration sometimes closes spontaneously or can be closed via catheter at a later time ^64^.

Hemodynamically significant obstruction in the Fontan pathway may occur, most commonly in the left pulmonary artery ^57, 65^. In the absence of a prepulmonary pump, this can severely affect the Fontan circulation, and obstructions are routinely dilated or stented.

Systemic to pulmonary venous collaterals can produce a right-to-left shunting which often worsens in time. Coiling of these collaterals is routinely performed in some centers, although no survival benefit has been demonstrated ^66^.

Aortopulmonary collaterals are common in the Fontan circulation. They increase pulmonary blood flow but induce a volume overload on the SV and might increase pulmonary artery pressure, limiting flow from the caval veins ^65^. During exercise, aortopulmonary flow increases, possibly augmenting loading conditions of the ventricle ^67^. A large aortopulmonary collateral burden has been associated with worse short-term outcomes ^68^. No clear consensus regarding the long-term effects and management of these collaterals exists ^65^.

Catheter ablation of an arrhythmogenic substrate is common. Long-term success rates vary between 15 and 72% ^69, 70^.

The reported incidence of catheter-based interventions (that is, excluding diagnostic cardiac catheterization without intervention) varies heavily; 3 to 65% of patients require at least one additional catheter intervention following Fontan completion ^31, 56, 57, 59, 71^. The most common catheter interventions are fenestration closure (10 to 64% of patients with a fenestration require catheter-based fenestration closing ^56, 57, 59, 72^), occlusion of veno-venous or aortopulmonary collaterals (incidence of 10 to 20%) ^57, 59^, and stenting and dilation of (all types of) obstructions in the Fontan pathway (incidence of 6 to 19%) ^57, 59, 73^.

### Other outcomes and functional status

Most studies report a diminished quality of life in Fontan patients compared with healthy controls ^74– 78^. Physical and emotional functioning are the most severely affected domains ^74, 79^. Low perceived health status can lead to unnecessary restrictions in daily life. Furthermore, increased rates of developmental disorders and lower intelligence scores have been reported in the Fontan population ^53, 76^.

Fontan patients have a moderately decreased exercise capacity compared with healthy controls ^80^. Mean peak oxygen uptake ranges from 61 to 74% of predicted values ^80– 82^. A small fraction of Fontan patients have normal exercise capacity ^83^. Exercise capacity in the Fontan patient has been shown to decrease over time ^81, 82^. Exercise capacity is predictive of hospital admissions, quality of life, and late mortality ^25, 28, 79, 82^. Exercise training can be done successfully in Fontan patients and can improve quality of life, functional class, and health perception in a short-term follow-up ^84– 87^. Whether exercise training has a role in optimizing long-term outcome is currently not clear ^84, 86– 89^. Resistance training can be used to increase muscle mass. In the Fontan patient, this could augment peripheral venous return, augment ventricular preload, and improve cardiac output ^90^. Similarly, a benefit of inspiratory muscle training has been demonstrated ^91^.

Despite high morbidity and suboptimal outcomes, most patients with a well-functioning Fontan circulation manage to lead fulfilling lives, are employed, may attain academic achievements, can participate in sports, and are able to successfully carry pregnancy to term ^92– 94^.

### Assessment techniques

Fontan patients are routinely assessed for health and functional status. Diagnostic cardiac catheterization has been standard practice in the pre-TCPC evaluation, as it provides excellent anatomic and necessary hemodynamic information regarding the pulmonary artery pressure, pulmonary vascular resistance, and end-diastolic SV pressure ^95^. There is recent interest in omitting cardiac catheterization in the pre-TCPC assessment for low-risk SV patients ^95– 97^. No consensus regarding this policy has been reached, as long-term outcomes are currently unavailable. Catheter-based interventions are discussed above.

Cardiac magnetic resonance imaging (CMR) is routinely performed during follow-up after TCPC, particularly to assess ventricular size and function and to quantify large vessel flow, including the amount of collateral flow ^98– 102^. Death and (being listed for) heart transplantation have been associated with higher end-diastolic volume index (EDVi) (>125 mL/m ^2^) as assessed with CMR in adolescents with a Fontan circulation ^103, 104^. Combined with a computational fluid dynamics approach, CMR might provide very useful information on the Fontan circulation and can aid in the evaluation of modifications in treatment strategies ^104, 105^.

Echocardiographic strain measurements have been shown to predict survival in the Fontan population and predict length of hospital stay following TCPC ^104, 106^. Assessment of ventriculo-arterial (VA) coupling may be another important parameter, as it is independent of the (often impaired) ventricular load. VA coupling has been shown to be suboptimal in some Fontan populations ^107^. Currently, VA coupling has not been associated with long-term outcomes in the Fontan population.

Lymphangiography could play an important role in the management of Fontan complications with a suspected lymphatic pathogenesis, such as PLE and plastic bronchitis. In patients with PLE or plastic bronchitis, increased diameters of major lymphatic vessels have been noted ^108^. Abnormal lymphatic depositions in the lungs and liver have been visualized in patients with plastic bronchitis and PLE, respectively ^109, 110^.

### Medical therapy

Anticoagulation, in the form of anti-platelet drugs or vitamin K antagonists (VKAs), is commonly indicated considering the increased risk for thromboembolic events, as discussed above in the “Extracardiac complications” section ^111^. A meta-analysis by Alsaied *et al*. showed that both acetylsalicylic acid and VKA were equally effective in preventing thromboembolic complications ^112^. However, if international normalized ratio (INR) is not properly controlled, outcomes on VKA are worse compared with acetylsalicylic acid ^113^. Novel oral anticoagulants (NOACs) do not require frequent monitoring and have mostly outperformed VKA in the adult population. Thirty-day outcomes following NOAC initiation show no major adverse events in the adult CHD population ^114^. However, no NOAC agent currently has US Food and Drug Administration approval for use in children.

### Medical prevention of circulatory failure in the Fontan circulation

Various medications have been assessed in the management of Fontan failure. No studies have shown benefit of angiotensin-converting enzyme (ACE) inhibitor therapy on survival, ventricular function, or cardiopulmonary exercise outcomes ^115, 116^.

Vasodilator drugs have been used to lower pulmonary vascular resistance ^117, 118^. Sildenafil has increased ventricular function, exercise capacity, and New York Heart Association (NYHA) status after 6 weeks of follow-up ^119, 120^. The effects of bosentan, an endothelin antagonist, in the Fontan population have varied ^121– 126^. No long-term survival benefit of vasodilator therapy has yet been demonstrated ^119^.

### Mechanical circulatory support for the failing Fontan circulation

The failing Fontan circulation can be supported by mechanical assist devices. Despite increasing use and the development of novel devices specifically for the pediatric and CHD population, experience in this population is still limited ^127^. Mechanical support devices are mostly used as a bridge to transplant in the failing Fontan ^128^. Recent reports showed a 60% 12-month survival in 48 Fontan patients with a ventricular assist device, proving viability of longer mechanical circulatory support ^129, 130^. A total biventricular artificial heart, the SynCardia, has been used to bridge a failing Fontan patient to transplant ^131^. A registry of mechanical circulatory support specifically for SV patients has been initiated ^132^. Currently, mechanical circulatory support in Fontan patients is associated with worse survival compared with mechanical circulatory support patients with a biventricular circulation ^133– 135^.

### Cardiac transplantation

Cardiac transplantation is the only treatment that truly corrects Fontan physiology, and it is employed in the failing Fontan circulation. In large cohorts, 1.6 to 3.6% of patients ultimately underwent cardiac transplant ^23, 24^. Survival following cardiac transplantation in Fontan patients is generally worse compared with other types of CHD ^136, 137^. Five-year survival ranges from 60 to 67% ^136– 138^.

### Surgical innovations

Continual efforts are made to improve the surgical techniques used in Fontan surgery. Recently, a Y-shaped graft was proposed for the connection of the inferior vena cava to the left and right pulmonary artery ^139^. Theoretically, this graft is more energetically favorable and provides better distribution of hepatic blood flow between the left and right pulmonary artery, distributing “hepatic factors” that may prevent the formation of intrapulmonary collaterals more equally. Worse energetic performance and pulmonary flow distributions in comparison with ECC connections have been noticed in practice ^140, 141^.

Fontan completion without cardiopulmonary bypass, particularly with the ECC technique, is an attractive option. However, experience is still limited and reported rates of conduit replacement and outcomes following off-pump procedures differ across centers ^142– 145^.

Less-invasive surgical approaches such as lateral thoracotomy have been described in this population ^146^. Hybrid procedures, which combine transcatheter and surgical approaches, have been implemented in the initial management of HLHS ^147^. Long-term outcomes are favorable, and some centers have adopted this hybrid approach as the standard for selected patient populations ^148^.

## Remaining challenges

A contemporary Fontan strategy uses either the ILT or the ECC modification. Two large meta-analyses have recently compared surgical strategies and found no differences in early or late mortality and Fontan takedown between ECC and ILT ^33, 149^. Theoretical advantages of both techniques have been discussed extensively in the literature ^150, 151^. Further research should assess contemporary differences in outcomes between modifications and help guide the preferred procedure for future Fontan patients. This may include alternative concepts, like the Y-graft or combinations with external energy supply (pumps).

Remodeling of the SV, which is exposed to volume overload at birth and is volume-deprived following the TCPC procedure, is not very well understood. A better understanding of mechanisms of remodeling during these stages and the interaction of ventricular size and function with the Fontan baffle function, pulmonary circulation, atrial function, and VA interaction is required to find better means to preserve cardiac function. The search for new targets for drugs that may help to preserve cardiac and circulatory function continues.

Some controversy regarding the timing of TCPC surgery exists. Proponents of early TCPC argue that a prolonged period of volume overload leads to adverse cardiac remodeling and reduced cardiac function ^152^. Others argue that the Fontan circulation inherently leads to complications and that surgery should be delayed to reduce the amount of time in Fontan physiology ^153^. Other factors to be considered are the techniques used; ILT allows TCPC at lower body weight than ECC since small-sized conduits (<18 mm) need to be avoided ^152, 154^. Studies assessing the optimal timing of ECC procedures are currently being performed.

The effect of systemic to pulmonary venous and aortopulmonary collaterals on the Fontan circulation remains poorly understood. These collaterals could provide some benefit in patients with a suboptimal Fontan circuit. How these collaterals develop and why some patients seem more prone to this development remain to be determined. Increasing our understanding of the role of collaterals could help guide the selection of patients who will benefit from intervention. This requires well-designed (multicenter) studies. Several treatment modalities of PLE have been described in small series, including catheter-based strategies of both blood and lymphatic vessels and surgical re-implantation of the innominate vein into the common atrium ^155– 159^. More comprehensive analysis is needed to determine the efficacy and safety of these procedures.

Drug therapy has been shown to be able to decrease pulmonary vascular resistance in the short-term, making this a promising therapy for the Fontan patient. However, currently, no long-term benefit has been demonstrated. The role of drug therapy in the Fontan circulation needs to be studied more extensively.

These questions require answers to make better-informed decisions in the management of these challenging patients, who have some of the most severe kinds of CHD. We have an opportunity to help this growing patient population not just to survive but also to thrive and live full and satisfying lives.

## Conclusions

The modern Fontan strategy has significantly transformed outcomes for patients with univentricular CHD. This has led to a large and growing population of Fontan patients surviving into adulthood. However, morbidity remains high and increases as this population ages and grows in proportion. Efforts to reduce morbidity and improve quality of life in these patients are ongoing. These efforts are focused on improving surgical techniques, developing novel diagnostic and therapeutic tools, and increasing our understanding of the highly abnormal Fontan physiology.

## Abbreviations

APC, atriopulmonary connection; AV, atrioventricular; CHD, congenital heart disease; CMR, cardiac magnetic resonance (imaging); ECC, extracardiac conduit; GFR, glomerular filtration rate; HLHS, hypoplastic left heart syndrome; ILT, intra-atrial lateral tunnel; MRI, magnetic resonance imaging; NOAC, novel oral anticoagulant; PCPC, partial cavopulmonary connection; PLE, protein-losing enteropathy; SV, single ventricle; SVT, supraventricular tachycardia; TCPC, total cavopulmonary connection; VA, ventricular-arterial; VKA, vitamin K antagonist.

## References

[1] Idorn L Olsen M Jensen AS: Univentricular hearts in Denmark 1977 to 2009: incidence and survival. *Int J Cardiol.* 2013;167(4):1311–6. 10.1016/j.ijcard.2012.03.182 22521378

[2] Moons P Sluysmans T De Wolf D: Congenital heart disease in 111 225 births in Belgium: birth prevalence, treatment and survival in the 21st century. *Acta Paediatr.* 2009;98(3):472–7. 10.1111/j.1651-2227.2008.01152.x 19046347

[3] Schwedler G Lindinger A Lange PE: Frequency and spectrum of congenital heart defects among live births in Germany: a study of the Competence Network for Congenital Heart Defects. *Clin Res Cardiol.* 2011;100(12):1111–7. 10.1007/s00392-011-0355-7 21909849

[4] Khairy P Poirier N Mercier LA: Univentricular heart. *Circulation.* 2007;115(6):800–12. 10.1161/CIRCULATIONAHA.105.592378 17296869

[5] Coats L O'Connor S Wren C: The single-ventricle patient population: a current and future concern a population-based study in the North of England. *Heart.* 2014;100(17):1348–53. 10.1136/heartjnl-2013-305336 24794141

[6] Beghetti M: Pulmonary vasodilators in Fontan Patients. EUROGUCH 2017; Lausanne2017 Reference Source

[7] van Velzen CL Ket JCF van de Ven PM: Systematic review and meta-analysis of the performance of second-trimester screening for prenatal detection of congenital heart defects. *Int J Gynaecol Obstet.* 2018;140(2):137–45. 10.1002/ijgo.12373 29094357

[8] Fontan F Baudet E: Surgical repair of tricuspid atresia. *Thorax.* 1971;26(3):240–8. 10.1136/thx.26.3.240 5089489 PMC1019078

[9] Poh CL Zannino D Weintraub RG: Three decades later: The fate of the population of patients who underwent the Atriopulmonary Fontan procedure. *Int J Cardiol.* 2017;231:99–104. 10.1016/j.ijcard.2017.01.057 28100430

[10] de Leval MR Kilner P Gewillig M: Total cavopulmonary connection: a logical alternative to atriopulmonary connection for complex Fontan operations. Experimental studies and early clinical experience. *J Thorac Cardiovasc Surg.* 1988;96(5):682–95. 3184963

[11] Kerlo AEM Delorme YT Xu D: Experimental characterization of powered Fontan hemodynamics in an idealized total cavopulmonary connection model. *Exp Fluids.* 2013;54:1581 10.1007/s00348-013-1581-8

[12] Huang L Dalziel KM Schilling C: Hospital costs and cost implications of co-morbid conditions for patients with single ventricle in the period through to Fontan completion. *Int J Cardiol.* 2017;240:178–82. 10.1016/j.ijcard.2017.04.056 28456482

[13] Cedars A Benjamin L Vyhmeister R: Contemporary Hospitalization Rate Among Adults With Complex Congenital Heart Disease. *World J Pediatr Congenit Heart Surg.* 2016;7(3):334–43. 10.1177/2150135116639541 27142401

[14] Tabtabai S DeFaria Yeh D Stefanescu A: National Trends in Hospitalizations for Patients With Single-Ventricle Anatomy. *Am J Cardiol.* 2015;116(5):773–8. 10.1016/j.amjcard.2015.05.053 26100589

[15] Collins RT 2nd Fram RY Tang X: Hospital utilization in adults with single ventricle congenital heart disease and cardiac arrhythmias. *J Cardiovasc Electrophysiol.* 2014;25(2):179–86. 10.1111/jce.12294 24102747

[16] Huang L Schilling C Dalziel KM: Hospital Inpatient Costs for Single Ventricle Patients Surviving the Fontan Procedure. *Am J Cardiol.* 2017;120(3):467–72. 10.1016/j.amjcard.2017.04.049 28583678

[17] Gewillig M: The Fontan circulation. *Heart.* 2005;91(6):839–46. 10.1136/hrt.2004.051789 15894794 PMC1768934

[18] Rychik J: The Relentless Effects of the Fontan Paradox. *Semin Thorac Cardiovasc Surg Pediatr Card Surg Annu.* 2016;19(1):37–43. 10.1053/j.pcsu.2015.11.006 27060041

[19] Fontan F Kirklin JW Fernandez G: Outcome after a "perfect" Fontan operation. *Circulation.* 1990;81(5):1520–36. 10.1161/01.CIR.81.5.1520 2331765

[20] Gewillig M Brown SC: The Fontan circulation after 45 years: update in physiology. *Heart.* 2016;102(14):1081–6. 10.1136/heartjnl-2015-307467 27220691 PMC4941188

[21] Kverneland LS Kramer P Ovroutski S: Five decades of the Fontan operation: A systematic review of international reports on outcomes after univentricular palliation. *Congenit Heart Dis.* 2018;13(2):181–93. 10.1111/chd.12570 29372588

[22] Iyengar AJ Winlaw DS Galati JC: Trends in Fontan surgery and risk factors for early adverse outcomes after Fontan surgery: the Australia and New Zealand Fontan Registry experience. *J Thorac Cardiovasc Surg.* 2014;148(2):566–75. 10.1016/j.jtcvs.2013.09.074 24280718

[23] d'Udekem Y Iyengar AJ Galati JC: Redefining expectations of long-term survival after the Fontan procedure: twenty-five years of follow-up from the entire population of Australia and New Zealand. *Circulation.* 2014;130(11 Suppl 1):S32–8. 10.1161/CIRCULATIONAHA.113.007764 25200053

[24] Pundi KN Johnson JN Dearani JA: 40-Year Follow-Up After the Fontan Operation: Long-Term Outcomes of 1,052 Patients. *J Am Coll Cardiol.* 2015;66(15):1700–10. 10.1016/j.jacc.2015.07.065 26449141

[25] Alsaied T Bokma JP Engel ME: Factors associated with long-term mortality after Fontan procedures: a systematic review. *Heart.* 2017;103(2):104–10. 10.1136/heartjnl-2016-310108 28057809

[26] Ohuchi H Yasuda K Miyazaki A: Comparison of prognostic variables in children and adults with Fontan circulation. *Int J Cardiol.* 2014;173(2):277–83. 10.1016/j.ijcard.2014.03.001 24650660

[27] Fernandes SM Alexander ME Graham DA: Exercise testing identifies patients at increased risk for morbidity and mortality following Fontan surgery. *Congenit Heart Dis.* 2011;6(4):294–303. 10.1111/j.1747-0803.2011.00500.x 21418537

[28] Diller GP Giardini A Dimopoulos K: Predictors of morbidity and mortality in contemporary Fontan patients: results from a multicenter study including cardiopulmonary exercise testing in 321 patients. *Eur Heart J.* 2010;31(24):3073–83. 10.1093/eurheartj/ehq356 20929979

[29] Assenza GE Graham DA Landzberg MJ: MELD-XI score and cardiac mortality or transplantation in patients after Fontan surgery. *Heart.* 2013;99(7):491–6. 10.1136/heartjnl-2012-303347 23406689

[30] Dimopoulos K Diller GP Koltsida E: Prevalence, predictors, and prognostic value of renal dysfunction in adults with congenital heart disease. *Circulation.* 2008;117(18):2320–8. 10.1161/CIRCULATIONAHA.107.734921 18443238

[31] Nakano T Kado H Tatewaki H: Results of extracardiac conduit total cavopulmonary connection in 500 patients. *Eur J Cardiothorac Surg.* 2015;48(6):825–32; discussion 832. 10.1093/ejcts/ezv072 25769469

[32] Giannakoulas G Dimopoulos K Yuksel S: Atrial tachyarrhythmias late after Fontan operation are related to increase in mortality and hospitalization. *Int J Cardiol.* 2012;157(2):221–6. 10.1016/j.ijcard.2010.12.049 21196055

[33] Lin Z Ge H Xue J: Comparison of extracardiac conduit and lateral tunnel for functional single-ventricle patients: A meta-analysis. *Congenit Heart Dis.* 2017;12(6):711–20. 10.1111/chd.12503 28845580

[34] Bossers SS Duppen N Kapusta L: Comprehensive rhythm evaluation in a large contemporary Fontan population. *Eur J Cardiothorac Surg.* 2015;48(6):833–40; discussion 840–1. 10.1093/ejcts/ezu548 25602059

[35] Atz AM Zak V Mahony L: Longitudinal Outcomes of Patients With Single Ventricle After the Fontan Procedure. *J Am Coll Cardiol.* 2017;69(22):2735–44. 10.1016/j.jacc.2017.03.582 28571639 PMC5604334

[36] Deal BJ Jacobs ML: Management of the failing Fontan circulation. *Heart.* 2012;98(14):1098–104. 10.1136/heartjnl-2011-301133 22739639 PMC3386650

[37] Viswanathan S: Thromboembolism and anticoagulation after Fontan surgery. *Ann Pediatr Cardiol.* 2016;9(3):236–40. 10.4103/0974-2069.189109 27625521 PMC5007932

[38] Egbe AC Connolly HM McLeod CJ: Thrombotic and Embolic Complications Associated With Atrial Arrhythmia After Fontan Operation: Role of Prophylactic Therapy. *J Am Coll Cardiol.* 2016;68(12):1312–9. 10.1016/j.jacc.2016.06.056 27634123

[39] Seipelt RG Franke A Vazquez-Jimenez JF: Thromboembolic complications after Fontan procedures: comparison of different therapeutic approaches. *Ann Thorac Surg.* 2002;74(2):556–62. 10.1016/S0003-4975(02)03677-9 12173844

[40] Egbe AC Connolly HM Niaz T: Prevalence and outcome of thrombotic and embolic complications in adults after Fontan operation. *Am Heart J.* 2017;183:10–7. 10.1016/j.ahj.2016.09.014 27979032

[41] Balling G Vogt M Kaemmerer H: Intracardiac thrombus formation after the Fontan operation. *J Thorac Cardiovasc Surg.* 2000;119(4 Pt 1):745–52. 10.1016/S0022-5223(00)70010-9 10733763

[42] Khairy P Fernandes SM Mayer JE: Long-term survival, modes of death, and predictors of mortality in patients with Fontan surgery. *Circulation.* 2008;117(1):85–92. 10.1161/CIRCULATIONAHA.107.738559 18071068

[43] Ghaferi AA Hutchins GM: Progression of liver pathology in patients undergoing the Fontan procedure: Chronic passive congestion, cardiac cirrhosis, hepatic adenoma, and hepatocellular carcinoma. *J Thorac Cardiovasc Surg.* 2005;129(6):1348–52. 10.1016/j.jtcvs.2004.10.005 15942576

[44] Greenway SC Crossland DS Hudson M: Fontan-associated liver disease: Implications for heart transplantation. *J Heart Lung Transplant.* 2016;35(1):26–33. 10.1016/j.healun.2015.10.015 26586487

[45] Hilscher MB Johnson JN Cetta F: Surveillance for liver complications after the Fontan procedure. *Congenit Heart Dis.* 2017;12(2):124–32. 10.1111/chd.12446 28140526

[46] Daniels CJ Bradley EA Landzberg MJ: Fontan-Associated Liver Disease: Proceedings from the American College of Cardiology Stakeholders Meeting, October 1 to 2, 2015, Washington DC. *J Am Coll Cardiol.* 2017;70(25):3173–94. 10.1016/j.jacc.2017.10.045 29268929

[47] Johnson JN Driscoll DJ O'Leary PW: Protein-losing enteropathy and the Fontan operation. *Nutr Clin Pract.* 2012;27(3):375–84. 10.1177/0884533612444532 22516942

[48] Schwartz I McCracken CE Petit CJ: Late outcomes after the Fontan procedure in patients with single ventricle: a meta-analysis. *Heart.* 2018; pii: heartjnl-2017-312807. 10.1136/heartjnl-2017-312807 29535229

[49] Atz AM Zak V Mahony L: Survival data and predictors of functional outcome an average of 15 years after the Fontan procedure: the pediatric heart network Fontan cohort. *Congenit Heart Dis.* 2015;10(1):E30–42. 10.1111/chd.12193 24934522 PMC4414014

[50] Caruthers RL Kempa M Loo A: Demographic characteristics and estimated prevalence of Fontan-associated plastic bronchitis. *Pediatr Cardiol.* 2013;34(2):256–61. 10.1007/s00246-012-0430-5 22797520 PMC3586576

[51] Lee D Levin A Kiess M: Chronic kidney damage in the adult Fontan population. *Int J Cardiol.* 2018;257:62–6. 10.1016/j.ijcard.2017.11.118 29506739

[52] Opotowsky AR Baraona FR Mc Causland FR: Estimated glomerular filtration rate and urine biomarkers in patients with single-ventricle Fontan circulation. *Heart.* 2017;103(6):434–42. 10.1136/heartjnl-2016-309729 27670967 PMC5500305

[53] Singh S Kumar R Roy B: Regional brain gray matter changes in adolescents with single ventricle heart disease. *Neurosci Lett.* 2018;665:156–62. 10.1016/j.neulet.2017.12.011 29222023 PMC5801206

[54] Pike NA Roy B Gupta R: Brain abnormalities in cognition, anxiety, and depression regulatory regions in adolescents with single ventricle heart disease. *J Neurosci Res.* 2018;96(6):1104–18. 10.1002/jnr.24215 29315714 PMC6103299

[55] Muneuchi J Nagatomo Y Okada S: Increased Pituitary Volumes in Children after Fontan Operation: Congestion in the Other Portal Circulation. *J Pediatr.* 2018;193:249–51. 10.1016/j.jpeds.2017.09.065 29198765

[56] Ono M Boethig D Goerler H: Clinical outcome of patients 20 years after Fontan operation--effect of fenestration on late morbidity. *Eur J Cardiothorac Surg.* 2006;30(6):923–9. 10.1016/j.ejcts.2006.08.025 17074498

[57] Hannan RL Zabinsky JA Salvaggio JL: The Fontan operation: the pursuit of associated lesions and cumulative trauma. *Pediatr Cardiol.* 2011;32(6):778–84. 10.1007/s00246-011-9973-0 21479823 PMC3139070

[58] Palumbo T Sluysmans T Rubay JE: Long-term outcome and anaesthetic management for non-cardiac surgery after Fontan palliation: a single-centre retrospective analysis. *Cardiol Young.* 2015;25(6):1148–54. 10.1017/S1047951114001814 25245855

[59] Downing TE Allen KY Goldberg DJ: Surgical and Catheter-Based Reinterventions Are Common in Long-Term Survivors of the Fontan Operation. *Circ Cardiovasc Interv.* 2017;10(9): pii: e004924. 10.1161/CIRCINTERVENTIONS.116.004924 28851719

[60] Honjo O Atlin CR Mertens L: Atrioventricular valve repair in patients with functional single-ventricle physiology: impact of ventricular and valve function and morphology on survival and reintervention. *J Thorac Cardiovasc Surg.* 2011;142(2):326–35.e2. 10.1016/j.jtcvs.2010.11.060 21592529

[61] Mavroudis C Deal BJ: Fontan Conversion: Literature Review and Lessons Learned Over 20 Years. *World J Pediatr Congenit Heart Surg.* 2016;7(2):192–8. 10.1177/2150135115623960 26957403

[62] Warnes CA Williams RG Bashore TM: ACC/AHA 2008 Guidelines for the Management of Adults with Congenital Heart Disease: Executive Summary: a report of the American College of Cardiology/American Heart Association Task Force on Practice Guidelines (writing committee to develop guidelines for the management of adults with congenital heart disease). *Circulation.* 2008;118(23):2395–451. 10.1161/CIRCULATIONAHA.108.190811 18997168

[63] Honjo O Mertens L van Arsdell GS: Atrioventricular valve repair in patients with single-ventricle physiology: mechanisms, techniques of repair, and clinical outcomes. *Semin Thorac Cardiovasc Surg Pediatr Card Surg Annu.* 2011;14(1):75–84. 10.1053/j.pcsu.2011.02.001 21444052

[64] Bridges ND Lock JE Castaneda AR: Baffle fenestration with subsequent transcatheter closure. Modification of the Fontan operation for patients at increased risk. *Circulation.* 1990;82(5):1681–9. 10.1161/01.CIR.82.5.1681 2225370

[65] Kreutzer J Graziano JN Stapleton G: Late catheter interventions in hypoplastic left heart syndrome. *Cardiol Young.* 2011;21Suppl 2:65–76. 10.1017/S1047951111001612 22152531

[66] Poterucha JT Johnson JN Taggart NW: Embolization of Veno-venous Collaterals after the Fontan Operation Is Associated with Decreased Survival. *Congenit Heart Dis.* 2015;10(5):E230–6. 10.1111/chd.12276 26010433

[67] Schmitt B Steendijk P Ovroutski S: Pulmonary vascular resistance, collateral flow, and ventricular function in patients with a Fontan circulation at rest and during dobutamine stress. *Circ Cardiovasc Imaging.* 2010;3(5):623–31. 10.1161/CIRCIMAGING.109.931592 20631032

[68] Odenwald T Quail MA Giardini A: Systemic to pulmonary collateral blood flow influences early outcomes following the total cavopulmonary connection. *Heart.* 2012;98(12):934–40. 10.1136/heartjnl-2011-301599 22626901

[69] de Groot NM Lukac P Blom NA: Long-term outcome of ablative therapy of postoperative supraventricular tachycardias in patients with univentricular heart: a European multicenter study. *Circ Arrhythm Electrophysiol.* 2009;2(3):242–8. 10.1161/CIRCEP.108.828137 19808474

[70] Yap S Harris L Silversides CK: Outcome of intra-atrial re-entrant tachycardia catheter ablation in adults with congenital heart disease: negative impact of age and complex atrial surgery. *J Am Coll Cardiol.* 2010;56(19):1589–96. 10.1016/j.jacc.2010.04.061 21029876

[71] Wilson TG Shi WY Iyengar AJ: Twenty-Five Year Outcomes of the Lateral Tunnel Fontan Procedure. *Semin Thorac Cardiovasc Surg.* 2017;29(3):347–353. 10.1053/j.semtcvs.2017.06.002 29195575

[72] Pihkala JI Järvelä M Boldt T: Fate of fenestration in children treated with fontan operation. *Catheter Cardiovasc Interv.* 2016;87(6):E233–9. 10.1002/ccd.26324 26525305

[73] Dancea A Justino H Martucci G: Catheter intervention for congenital heart disease at risk of circulatory failure. *Can J Cardiol.* 2013;29(7):786–795. 10.1016/j.cjca.2013.04.021 23790551

[74] Uzark K Zak V Shrader P: Assessment of Quality of Life in Young Patients with Single Ventricle after the Fontan Operation. *J Pediatr.* 2016;170:166–72.e1. 10.1016/j.jpeds.2015.11.016 26685073 PMC4769899

[75] Smaś-Suska M Dłużniewska N Weryński P: What determines the quality of life of adult patients after Fontan procedure? *Cardiol J.* 2018;25(1):72–80. 10.5603/CJ.a2017.0078 28695974

[76] Idorn L Jensen AS Juul K: Quality of life and cognitive function in Fontan patients, a population-based study. *Int J Cardiol.* 2013;168(4):3230–5. 10.1016/j.ijcard.2013.04.008 23632112

[77] Vahsen N Bröder A Hraska V: Neurodevelopmental Outcome in Children With Single Ventricle After Total Cavopulmonary Connection. *Klin Padiatr.* 2018;230(1):24–30. 10.1055/s-0043-120526 29258158

[78] Kukreja M Bryant AS Cleveland DC: Health-Related Quality of Life in Adult Survivors After the Fontan Operation. *Semin Thorac Cardiovasc Surg.* 2015;27(3):299–306. 10.1053/j.semtcvs.2015.08.007 26708372

[79] Dulfer K Bossers SS Utens EM: Does functional health status predict health-related quality of life in children after Fontan operation? *Cardiol Young.* 2016;26(3):459–68. 10.1017/S1047951115000426 25906441

[80] Bossers SS Helbing WA Duppen N: Exercise capacity in children after total cavopulmonary connection: lateral tunnel versus extracardiac conduit technique. *J Thorac Cardiovasc Surg.* 2014;148(4):1490–7. 10.1016/j.jtcvs.2013.12.046 24521957

[81] Giardini A Hager A Pace Napoleone C: Natural history of exercise capacity after the Fontan operation: a longitudinal study. *Ann Thorac Surg.* 2008;85(3):818–21. 10.1016/j.athoracsur.2007.11.009 18291148

[82] Egbe AC Driscoll DJ Khan AR: Cardiopulmonary exercise test in adults with prior Fontan operation: The prognostic value of serial testing. *Int J Cardiol.* 2017;235:6–10. 10.1016/j.ijcard.2017.02.140 28284501

[83] Cordina R Du Plessis K Tran D: Super-Fontan: Is it possible? *J Thorac Cardiovasc Surg.* 2018;155(3):1192–4. 10.1016/j.jtcvs.2017.10.047 29157925

[84] Dulfer K Duppen N Kuipers IM: Aerobic exercise influences quality of life of children and youngsters with congenital heart disease: a randomized controlled trial. *J Adolesc Health.* 2014;55(1):65–72. 10.1016/j.jadohealth.2013.12.010 24518533

[85] McCrindle BW Williams RV Mital S: Physical activity levels in children and adolescents are reduced after the Fontan procedure, independent of exercise capacity, and are associated with lower perceived general health. *Arch Dis Child.* 2007;92(6):509–14. 10.1136/adc.2006.105239 17307794 PMC2066169

[86] Duppen N Etnel JR Spaans L: Does exercise training improve cardiopulmonary fitness and daily physical activity in children and young adults with corrected tetralogy of Fallot or Fontan circulation? A randomized controlled trial. *Am Heart J.* 2015;170(3):606–14. 10.1016/j.ahj.2015.06.018 26385046

[87] Takken T Hulzebos HJ Blank AC: Exercise prescription for patients with a Fontan circulation: current evidence and future directions. *Neth Heart J.* 2007;15(4):142–7. 10.1007/BF03085970 17612674 PMC1847768

[88] Brassard P Bédard E Jobin J: Exercise capacity and impact of exercise training in patients after a Fontan procedure: a review. *Can J Cardiol.* 2006;22(6):489–95. 10.1016/S0828-282X(06)70266-5 16685313 PMC2560550

[89] Hedlund ER Lundell B Söderström L: Can endurance training improve physical capacity and quality of life in young Fontan patients? *Cardiol Young.* 2018;28(3):438–46. 10.1017/S1047951117002360 29237518

[90] Cordina RL O'Meagher S Karmali A: Resistance training improves cardiac output, exercise capacity and tolerance to positive airway pressure in Fontan physiology. *Int J Cardiol.* 2013;168(2):780–8. 10.1016/j.ijcard.2012.10.012 23154055

[91] Laohachai K Winlaw D Selvadurai H: Inspiratory Muscle Training Is Associated With Improved Inspiratory Muscle Strength, Resting Cardiac Output, and the Ventilatory Efficiency of Exercise in Patients With a Fontan Circulation. *J Am Heart Assoc.* 2017;6(8): pii: e005750. 10.1161/JAHA.117.005750 28862962 PMC5586429

[92] Khairy P Ouyang DW Fernandes SM: Pregnancy outcomes in women with congenital heart disease. *Circulation.* 2006;113(4):517–24. 10.1161/CIRCULATIONAHA.105.589655 16449731

[93] Mair DD Puga FJ Danielson GK: Late functional status of survivors of the Fontan procedure performed during the 1970s. *Circulation.* 1992;86(5 Suppl):II106–9. 1423987

[94] Pike NA Evangelista LS Doering LV: Clinical profile of the adolescent/adult Fontan survivor. *Congenit Heart Dis.* 2011;6(1):9–17. 10.1111/j.1747-0803.2010.00475.x 21269408 PMC3177559

[95] Mohammad Nijres B Murphy JJ Diab K: Routine Cardiac Catheterization Prior to Fontan Operation: Is It a Necessity? *Pediatr Cardiol.* 2018;39(4):818–23. 10.1007/s00246-018-1825-8 29396581

[96] Ro PS Rychik J Cohen MS: Diagnostic assessment before Fontan operation in patients with bidirectional cavopulmonary anastomosis: are noninvasive methods sufficient? *J Am Coll Cardiol.* 2004;44(1):184–7. 10.1016/j.jacc.2004.02.058 15234431

[97] Fogel MA Pawlowski TW Whitehead KK: Cardiac magnetic resonance and the need for routine cardiac catheterization in single ventricle patients prior to Fontan: a comparison of 3 groups: pre-Fontan CMR versus cath evaluation. *J Am Coll Cardiol.* 2012;60(12):1094–102. 10.1016/j.jacc.2012.06.021 22974693

[98] Valsangiacomo Buechel ER Grosse-Wortmann L Fratz S: Indications for cardiovascular magnetic resonance in children with congenital and acquired heart disease: an expert consensus paper of the Imaging Working Group of the AEPC and the Cardiovascular Magnetic Resonance Section of the EACVI. *Eur Heart J Cardiovasc Imaging.* 2015;16(3):281–97. 10.1093/ehjci/jeu129 25712078

[99] Glatz AC Rome JJ Small AJ: Systemic-to-pulmonary collateral flow, as measured by cardiac magnetic resonance imaging, is associated with acute post-Fontan clinical outcomes. *Circ Cardiovasc Imaging.* 2012;5(2):218–25. 10.1161/CIRCIMAGING.111.966986 22228054 PMC3310971

[100] Grosse-Wortmann L Drolet C Dragulescu A: Aortopulmonary collateral flow volume affects early postoperative outcome after Fontan completion: a multimodality study. *J Thorac Cardiovasc Surg.* 2012;144(6):1329–36. 10.1016/j.jtcvs.2012.03.032 22502974

[101] Lawley CM Broadhouse KM Callaghan FM: 4D flow magnetic resonance imaging: role in pediatric congenital heart disease. *Asian Cardiovasc Thorac Ann.* 2018;26(1):28–37. 10.1177/0218492317694248 28185475

[102] Bächler P Valverde I Pinochet N: Caval blood flow distribution in patients with Fontan circulation: quantification by using particle traces from 4D flow MR imaging. *Radiology.* 2013;267(1):67–75. 10.1148/radiol.12120778 23297331

[103] Rathod RH Prakash A Kim YY: Cardiac magnetic resonance parameters predict transplantation-free survival in patients with fontan circulation. *Circ Cardiovasc Imaging.* 2014;7(3):502–9. 10.1161/CIRCIMAGING.113.001473 24619103 PMC4262249

[104] Ghelani SJ Harrild DM Gauvreau K: Comparison Between Echocardiography and Cardiac Magnetic Resonance Imaging in Predicting Transplant-Free Survival After the Fontan Operation. *Am J Cardiol.* 2015;116(7):1132–8. 10.1016/j.amjcard.2015.07.011 26251003

[105] Whitehead KK Pekkan K Kitajima HD: Nonlinear power loss during exercise in single-ventricle patients after the Fontan: insights from computational fluid dynamics. *Circulation.* 2007;116(11 Suppl):I165–71. 10.1161/CIRCULATIONAHA.106.680827 17846299

[106] Park PW Atz AM Taylor CL: Speckle-Tracking Echocardiography Improves Pre-operative Risk Stratification Before the Total Cavopulmonary Connection. *J Am Soc Echocardiogr.* 2017;30(5):478–84. 10.1016/j.echo.2017.01.008 28274715 PMC5420476

[107] Saiki H Eidem BW Ohtani T: Ventricular-Arterial Function and Coupling in the Adult Fontan Circulation. *J Am Heart Assoc.* 2016;5(9): pii: e003887. 10.1161/JAHA.116.003887 27663413 PMC5079039

[108] Dori Y Keller MS Fogel MA: MRI of lymphatic abnormalities after functional single-ventricle palliation surgery. *AJR Am J Roentgenol.* 2014;203(2):426–31. 10.2214/AJR.13.11797 24848564

[109] Dori Y Keller MS Rome JJ: Percutaneous Lymphatic Embolization of Abnormal Pulmonary Lymphatic Flow as Treatment of Plastic Bronchitis in Patients With Congenital Heart Disease. *Circulation.* 2016;133(12):1160–70. 10.1161/CIRCULATIONAHA.115.019710 26864093

[110] Itkin M Piccoli DA Nadolski G: Protein-Losing Enteropathy in Patients With Congenital Heart Disease. *J Am Coll Cardiol.* 2017;69(24):2929–37. 10.1016/j.jacc.2017.04.023 28619193

[111] Cromme-Dijkhuis AH Hess J Hählen K: Specific sequelae after Fontan operation at mid- and long-term follow-up. Arrhythmia, liver dysfunction, and coagulation disorders. *J Thorac Cardiovasc Surg.* 1993;106:1126–32. 8246550

[112] Alsaied T Alsidawi S Allen CC: Strategies for thromboprophylaxis in Fontan circulation: a meta-analysis. *Heart.* 2015;101(21):1731–7. 10.1136/heartjnl-2015-307930 26319122

[113] McCrindle BW Manlhiot C Cochrane A: Factors associated with thrombotic complications after the Fontan procedure: a secondary analysis of a multicenter, randomized trial of primary thromboprophylaxis for 2 years after the Fontan procedure. *J Am Coll Cardiol.* 2013;61(3):346–53. 10.1016/j.jacc.2012.08.1023 23246393

[114] Yang H Bouma BJ Mulder BJM: Is Initiating NOACs for Atrial Arrhythmias Safe in Adults with Congenital Heart Disease? *Cardiovasc Drugs Ther.* 2017;31(4):413–7. 10.1007/s10557-017-6745-y 28785894 PMC5591797

[115] Wilson TG Iyengar AJ Winlaw DS: Use of ACE inhibitors in Fontan: Rational or irrational? *Int J Cardiol.* 2016;210:95–9. 10.1016/j.ijcard.2016.02.089 26938683

[116] Wilson TG Iyengar AJ d'Udekem Y: The Use and Misuse of ACE Inhibitors in Patients with Single Ventricle Physiology. *Heart Lung Circ.* 2016;25(3):229–36. 10.1016/j.hlc.2015.10.005 26775546

[117] Morchi GS Ivy DD Duster MC: Sildenafil Increases Systemic Saturation and Reduces Pulmonary Artery Pressure in Patients with Failing Fontan Physiology. *Congenit Heart Dis.* 2009;4(2):107–11. 10.1111/j.1747-0803.2008.00237.x 21866231 PMC3159127

[118] Giordano R Palma G Poli V: First experience with sildenafil after Fontan operation: short-term outcomes. *J Cardiovasc Med (Hagerstown).* 2015;16(8):552–5. 10.2459/JCM.0b013e328361390a 23588032

[119] Oldenburger NJ Mank A Etnel J: Drug therapy in the prevention of failure of the Fontan circulation: a systematic review. *Cardiol Young.* 2016;26(5):842–50. 10.1017/S1047951115002747 26947621

[120] Mori H Park IS Yamagishi H: Sildenafil reduces pulmonary vascular resistance in single ventricular physiology. *Int J Cardiol.* 2016;221:122–7. 10.1016/j.ijcard.2016.06.322 27400308

[121] Hebert A Mikkelsen UR Thilen U: Bosentan improves exercise capacity in adolescents and adults after Fontan operation: the TEMPO (Treatment With Endothelin Receptor Antagonist in Fontan Patients, a Randomized, Placebo-Controlled, Double-Blind Study Measuring Peak Oxygen Consumption) study. *Circulation.* 2014;130(23):2021–30. 10.1161/CIRCULATIONAHA.113.008441 25446057

[122] Shang X Lu R Zhang X: Efficacy of Bosentan in patients after Fontan procedures: a double-blind, randomized controlled trial. *J Huazhong Univ Sci Technol Med Sci.* 2016;36(4):534–40. 10.1007/s11596-016-1621-8 27465329

[123] Ovaert C Thijs D Dewolf D: The effect of bosentan in patients with a failing Fontan circulation. *Cardiol Young.* 2009;19(4):331–9. 10.1017/S1047951109990023 19519964

[124] Schuuring MJ Vis JC van Dijk AP: Impact of bosentan on exercise capacity in adults after the Fontan procedure: a randomized controlled trial. *Eur J Heart Fail.* 2013;15(6):690–8. 10.1093/eurjhf/hft017 23361871

[125] Park I: Efficacy of pulmonary vasodilator therapy in patients with functionally single ventricle. *Int Heart J.* 2015;56Suppl:S26–30. 10.1536/ihj.14-392 25787795

[126] Agnoletti G Gala S Ferroni F: Endothelin inhibitors lower pulmonary vascular resistance and improve functional capacity in patients with Fontan circulation. *J Thorac Cardiovasc Surg.* 2017;153(6):1468–75. 10.1016/j.jtcvs.2017.01.051 28283234

[127] Chopski SG Moskowitz WB Stevens RM: Mechanical Circulatory Support Devices for Pediatric Patients With Congenital Heart Disease. *Artif Organs.* 2017;41(1):E1–E14. 10.1111/aor.12760 27859378

[128] Carlo WF Villa CR Lal AK: Ventricular assist device use in single ventricle congenital heart disease. *Pediatr Transplant.* 2017;21(7):e13031. 10.1111/petr.13031 28921937

[129] Blume ED VanderPluym C Lorts A: Second annual Pediatric Interagency Registry for Mechanical Circulatory Support (Pedimacs) report: Pre-implant characteristics and outcomes. *J Heart Lung Transplant.* 2018;37(1):38–45. 10.1016/j.healun.2017.06.017 28965736

[130] Lorts A Eghtesady P Mehegan M: Outcomes of children supported with devices labeled as "temporary" or short term: A report from the Pediatric Interagency Registry for Mechanical Circulatory Support. *J Heart Lung Transplant.* 2018;37(1):54–60. 10.1016/j.healun.2017.10.023 29174220

[131] Rossano JW Goldberg DJ Fuller S: Successful use of the total artificial heart in the failing Fontan circulation. *Ann Thorac Surg.* 2014;97(4):1438–40. 10.1016/j.athoracsur.2013.06.120 24694426

[132] Rossano JW Woods RK Berger S: Mechanical support as failure intervention in patients with cavopulmonary shunts (MFICS): rationale and aims of a new registry of mechanical circulatory support in single ventricle patients. *Congenit Heart Dis.* 2013;8(3):182–6. 10.1111/chd.12053 23510301

[133] Poh CL Chiletti R Zannino D: Ventricular assist device support in patients with single ventricles: the Melbourne experience. *Interact Cardiovasc Thorac Surg.* 2017;25(2):310–6. 10.1093/icvts/ivx066 28486624

[134] Horne D Conway J Rebeyka IM: Mechanical circulatory support in univentricular hearts: current management. *Semin Thorac Cardiovasc Surg Pediatr Card Surg Annu.* 2015;18(1):17–24. 10.1053/j.pcsu.2015.02.002 25939838

[135] Weinstein S Bello R Pizarro C: The use of the Berlin Heart EXCOR in patients with functional single ventricle. *J Thorac Cardiovasc Surg.* 2014;147(2):697–704; discussion 70–45. 10.1016/j.jtcvs.2013.10.030 24290716

[136] Mauchley DC Mitchell MB: Transplantation in the Fontan patient. *Semin Thorac Cardiovasc Surg Pediatr Card Surg Annu.* 2015;18(1):7–16. 10.1053/j.pcsu.2015.01.001 25939837

[137] Rossano JW Shaddy RE: Heart transplant after the Fontan operation. *Cardiol Young.* 2013;23(6):841–6. 10.1017/S1047951113001662 24401256

[138] Kanter KR: Heart Transplantation in Children after a Fontan Procedure: Better than People Think. *Semin Thorac Cardiovasc Surg Pediatr Card Surg Annu.* 2016;19(1):44–9. 10.1053/j.pcsu.2015.11.004 27060042

[139] Martin MH Feinstein JA Chan FP: Technical feasibility and intermediate outcomes of using a handcrafted, area-preserving, bifurcated Y-graft modification of the Fontan procedure. *J Thorac Cardiovasc Surg.* 2015;149(1):239–45.e1. 10.1016/j.jtcvs.2014.08.058 25439786

[140] Trusty PM Wei Z Tree M: Local Hemodynamic Differences Between Commercially Available Y-Grafts and Traditional Fontan Baffles Under Simulated Exercise Conditions: Implications for Exercise Tolerance. *Cardiovasc Eng Technol.* 2017;8(3):390–9. 10.1007/s13239-017-0310-5 28560706

[141] Trusty PM Restrepo M Kanter KR: A pulsatile hemodynamic evaluation of the commercially available bifurcated Y-graft Fontan modification and comparison with the lateral tunnel and extracardiac conduits. *J Thorac Cardiovasc Surg.* 2016;151(6):1529–36. 10.1016/j.jtcvs.2016.03.019 27056758

[142] Mainwaring RD Reddy VM Hanley FL: Completion of the Three-Stage Fontan Pathway Without Cardiopulmonary Bypass. *World J Pediatr Congenit Heart Surg.* 2014;5(3):427–33. 10.1177/2150135114536908 24958046

[143] LaPar DJ Mery CM Peeler BB: Short and long-term outcomes for bidirectional glenn procedure performed with and without cardiopulmonary bypass. *Ann Thorac Surg.* 2012;94(1):164–70; discussion 170–1. 10.1016/j.athoracsur.2012.03.005 22560969

[144] Ovroutski S Sohn C Miera O: Improved early postoperative outcome for extracardiac Fontan operation without cardiopulmonary bypass: a single-centre experience. *Eur J Cardiothorac Surg.* 2013;43(5):952–7. 10.1093/ejcts/ezs535 23111560

[145] Talwar S Muthukkumaran S Choudhary SK: A complete extracorporeal circulation-free approach to patients with functionally univentricular hearts provides superior early outcomes. *World J Pediatr Congenit Heart Surg.* 2014;5(1):54–9. 10.1177/2150135113507091 24403355

[146] Sett SS Lafaro RJ: Extracardiac Fontan Operation Through a Right Thoracotomy. *Ann Thorac Surg.* 2017;104(2):e147–e149. 10.1016/j.athoracsur.2017.03.017 28734438

[147] Akintuerk H Michel-Behnke I Valeske K: Stenting of the arterial duct and banding of the pulmonary arteries: basis for combined Norwood stage I and II repair in hypoplastic left heart. *Circulation.* 2002;105(9):1099–103. 10.1161/hc0902.104709 11877362

[148] Schranz D Bauer A Reich B: Fifteen-year single center experience with the "Giessen Hybrid" approach for hypoplastic left heart and variants: current strategies and outcomes. *Pediatr Cardiol.* 2015;36(2):365–73. 10.1007/s00246-014-1015-2 25179460 PMC4303711

[149] Zheng J Li Z Li Q: Meta-analysis of Fontan procedure: Extracardiac conduit vs. intracardiac lateral tunnel. *Herz.* 2018;43(3):238–45. 10.1007/s00059-017-4553-6 28341981

[150] Kogon B: Is the extracardiac conduit the preferred Fontan approach for patients with univentricular hearts? The extracardiac conduit is the preferred Fontan approach for patients with univentricular hearts. *Circulation.* 2012;126(21):2511–5; discussion 2515. 10.1161/CIRCULATIONAHA.111.076398 23169252

[151] Khairy P Poirier N: Is the extracardiac conduit the preferred Fontan approach for patients with univentricular hearts? The extracardiac conduit is not the preferred Fontan approach for patients with univentricular hearts. *Circulation.* 2012;126(21):2516–25; discussion 2525. 10.1161/CIRCULATIONAHA.111.075036 23169253

[152] Pace Napoleone C Oppido G Angeli E: Results of the modified Fontan procedure are not related to age at operation. *Eur J Cardiothorac Surg.* 2010;37(3):645–50. 10.1016/j.ejcts.2009.09.003 19800250

[153] d'Udekem Y Xu MY Konstantinov IE: The optimal age at Fontan procedure and the 'ticking clock' theory: do we have an answer? *Eur J Cardiothorac Surg.* 2011;39(1):144; author reply 144–5. 10.1016/j.ejcts.2010.04.006 20554215

[154] Dabal RJ Kirklin JK Kukreja M: The modern Fontan operation shows no increase in mortality out to 20 years: a new paradigm. *J Thorac Cardiovasc Surg.* 2014;148(6): 2517–23.e1. 10.1016/j.jtcvs.2014.07.075 25277471

[155] Devanagondi R Suntharos P Boyle GJ: Protein Losing Enteropathy After Cardiac Transplantation Successfully Treated by Stent Implantation. *World J Pediatr Congenit Heart Surg.* 2017;8(6): 754–757. 10.1177/2150135116658452 27549730

[156] Brizard CP Lane GK Alex G: Original Surgical Procedure for the Treatment of Protein-Losing Enteropathy in Fontan Patients: Report of Two Midterm Successes. *Circulation.* 2016;134(8):625–7. 10.1161/CIRCULATIONAHA.116.023424 27550969

[157] Kreutzer C Kreutzer G: The Lymphatic System: The Achilles Heel of the Fontan-Kreutzer Circulation. *World J Pediatr Congenit Heart Surg.* 2017;8(5):613–623. 10.1177/2150135117720685 28901223

[158] Menon S Chennapragada M Ugaki S: The Lymphatic Circulation in Adaptations to the Fontan Circulation. *Pediatr Cardiol.* 2017;38(5):886–892. 10.1007/s00246-017-1576-y 28210768

[159] Hraška V: Decompression of thoracic duct: new approach for the treatment of failing Fontan. *Ann Thorac Surg.* 2013;96(2):709–11. 10.1016/j.athoracsur.2013.02.046 23910125

